# Claiming Positive Results From Negative Trials: A Cause for Concern in Randomized Controlled Trial Research - Author's Reply

**DOI:** 10.2196/jmir.2884

**Published:** 2013-08-19

**Authors:** Reid Hester, William Campbell, Kathryn Lenberg, Harold Delaney

**Affiliations:** ^1^Behavior Therapy Associates, LLCResearch Div.Albuquerque, NMUnited States; ^2^Presbyterian Medical GroupAlbuquerque, NMUnited States; ^3^University of New MexicoDept. of PsychologyAlbuquerque, NMUnited States

**Keywords:** addictions, cognitive-behavioral program, Web application, SMART Recovery, mutual self-help groups

## In Response

Cunningham is correct in noting that it is difficult to draw conclusions about the results of a randomized clinical trial (RCT) when two or more active interventions are compared without utilizing a no-treatment control condition. This is an issue that bedevils clinical research, but it is also one that, ethically speaking, has long been resolved in the area of addictions. As noted in the *New England Journal of Medicine*, when evaluating the risk-benefit ratio of employing a placebo or no-treatment control, potential psychological and social harms must be addressed [1]. The participants in this study comprised some of the most severe drinkers in our 20-plus years of conducting RCTs of computer delivered interventions for problem drinkers. Moreover, in this case, we were recruiting individuals in the contemplation and active stages of change, a window of opportunity well recognized among clinicians as the time when an intervention has the best chance of helping. Since treatment-seeking individuals struggling with serious alcohol disorders may be harmed by temporary conditions, such as a wait-list, the ethical criterion of doing no harm would not be met. Thus the clinically relevant question for us clearly became not whether a new treatment is better than nothing, but whether it is better than another treatment.

Given this limitation, it is incumbent upon researchers to be clear about their methods, conservative in their analysis, and parsimonious in their conclusions. To this end, we were clear when addressing our limitations that the lack of a no-treatment comparison group “prevents us from being assured that the treatment assigned was the cause of the improvement.” Cunningham is correct in observing that, as in virtually all clinical trials involving active interventions, alternative explanations may possibly account for the changes we observed in this trial. He mentions, in particular, client motivation and regression to the mean, so we will address those two concerns here.

While natural recovery does occur for many people with alcohol problems, it typically does so for those who tend to be at the less severe end of the spectrum [2]. As we noted, that would not likely pertain to the sample studied here, whose mean AUDIT scores were 24.7 and InDuC scores were 41.4.  It is relevant to note that the mean within-group effect size observed in no-treatment control groups is typically much smaller than the *d* of .97 seen in the OA+SR group or .96 in the SR only group in our study.  For example, the comparable mean pre-post effect sizes in five control groups reported in White et al. [3] ranged from -.61 to +.22 for drinks consumed with an overall mean weighted by study size of only *d* = +.05.  Thus, while other explanations of the cause of the effects we observed are conceivable in theory, in practice the implemented treatments seemed the most plausible explanation for the large effects observed.

As for the participants’ level of motivation coming in to the study, we were clear in our methods that we recruited from individuals who were actively seeking treatment options, and in so doing had arrived at the SMART Recovery website. This does bespeak of motivation, it’s hard to deny; but it is also hard to argue that we would have obtained the results we did in this clinical trial on the basis of motivation alone. It is a clinical truism that behavioral interventions require motivation on the part of patients to be effective. To that end, our results showed that whether participants in the trial received that intervention from SMART Recovery groups or from the Overcoming Addictions app, they generally succeeded in making significant reductions in their drinking and alcohol-related problems. That the data did not support better outcomes in one group or the other means, to us, that problem drinkers looking for help becoming and remaining abstinent, have options which are equally effective. And indeed, this is all we claim in our conclusions.

Cunningham characterizes the outcomes of our RCT as a “negative” trial and concludes that it is “unwise to favour an intervention effect explanation over other causes when faced with the results of an RCT where participants show improvement over time but that there are no significant differences between intervention conditions.” While the lack of difference between conditions does in fact remain a topic of empirical interest, we find his use of the term “negative” baffling, given the highly positive changes across both groups. We are also curious why Cunningham failed to raise these concerns earlier, with a plethora of other studies that compared active treatments without a no-treatment control [4, 5, 6, 7, 8] including his own [9].

Although we believe that Cunningham’s concerns with our conclusions are overblown, we welcome the opportunity to discuss the research methods of web-based interventions. Cunningham has argued convincingly elsewhere about the particular difficulties of research in this area [10], and to that end we strove as much as possible to generate results that would be generalizable. In this case developing an application specifically for individuals seeking help, and engaging in the intervention, on-line. Also, as per his recommendation [10], we sought to concurrently evaluate the effectiveness of SMART Recovery, the protocol upon which our application was based, since this had not been done previously.

Returning to the question of what factors mediate the effectiveness of two treatments under comparison, it might be interesting for Cunningham to explain how his own study [9], that found added benefits in a more involved intervention for problem drinkers, compared to Hansen et al.’s, findings which showed no such added benefit [8]. It is actually such questions as these that are more pressing for research into web-based interventions than are questions about the implications of not using a no-treatment control condition. As a field, we lack insight into these and other questions, such as why web-based interventions work in some instances and not others, why increased engagement leads to better outcomes in some cases and not others, and what sorts of individuals are most likely to benefit from web-based interventions. These are the sorts of data that other researchers in our field need, and it is just such data we will be reporting in Part 2 of our paper along with our six-month follow-up results.
